# Editorial: Omics biomarkers in inflammatory ocular diseases

**DOI:** 10.3389/fmed.2022.1000706

**Published:** 2022-08-12

**Authors:** Masaru Ttakeuchi, Mei Chen

**Affiliations:** ^1^Department of Ophthalmology, National Defense Medical College, Tokorozawa, Japan; ^2^The Wellcome-Wolfson Institute for Experimental Medicine, School of Medicine, Dentistry and Biomedical Sciences, Queen's University Belfast, Belfast, United Kingdom

**Keywords:** omics, ocular inflammation, inflammation, biomarker, omics biomarker

Rapid advances in biomedical technology allow us to collect different types of “omics” data with unprecedented details. Genome-wide data for various molecular processes, such as mRNA expression, DNA methylation, and microRNA (miRNA) expression, provides omics data for different disease studies. It seems to present only part of a complex biological reaction involved in the pathogenesis, but allows us to understand the underlying biological process where we had not reached (1–3). A great number of omics approaches have been proposed over the years, and a huge step forward was made by the increasing progression, specially designed for identification of biomarkers useful for early diagnostic and follow-up. In the Research Topic of Omics Biomarkers in Inflammatory Ocular Diseases, four outstanding omics studies have been submitted. Pterygium is an environmentally induced ocular surface degenerative disorder which may lead to blindness if untreated. Wolf et al. analyzed using MACE RNA sequencing and immunohistochemistry to characterize the transcriptional profile and the cellular microenvironment of conjunctival pterygia, and revealed genes associated with autophagy (including *DCN, TMBIM6*), cellular response to stress (including *TPT1, DDX5*) as well as fibroblast proliferation and epithelial to mesenchymal transition (including *CTNNB1, TGFBR1, and FN1*) were increased in pterygia compared to control tissue using RNA sequencing. These genes may be employed for new diagnostic tools and targeted therapeutic options for this common ocular surface disease. Blepharitis is a more common ocular surface disease, but is sometime intractable and the biomarkers have not been identified by omics for the pathogen. Wang et al. investigated the microbiota on the ocular surface of patients with blepharitis in northwestern China by 16S rDNA amplicon sequencing analysis. As a result, although the ocular surface microbiota of patients with blepharitis varied among different study groups, Lactobacillus, Bifidobacterium, Akkermansia, Ralstonia, and Bacteroides were identified as potential pathogens of blepharitis. On the other hand, superior limbic keratoconjunctivitis (SLK) is a bilateral, chronic inflammatory disease, and the pathogenic mechanisms remain unknown. Zong et al. performed metabolomic analysis using the tear fluids of SLK patients, and found that 31 metabolites significantly increased and 19 metabolites decreased in SLK patients, and indicated 9 metabolites (phenol, ethyl glucuronide, eicosapentaenoic acid, 12-keto-leukotriene B4, linoleic acid, hypoxanthine, triethanolamine, 1-nitrohexane, and terephthalic acid) as a candidate biomarker for SLK. Finally, Age-Related Macular Degeneration (AMD) is an increasing ocular disease worldwide, leading to irreversible vision impairment. In wet AMD, the progress of vision impairment has been able to be prevented by intravitreal injection of anti-vascular endothelial growth factor inhibitors, however for dry AMD, even a key molecule acting as the biomarker or the therapeutic target has not been identified yet. Mallik et al. focused on regression-based biological age clocks in the retina which have not yet been studied in AMD, and analyzed transcriptomic data consisting of a total of 453 retina samples including 105 Minnesota Grading System (MGS) level 1 samples, 175 MGS level 2, 112 MGS level 3 and 61 MGS level 4 samples, as well as 167 fibroblast samples. The clocks yielded good separation among AMD samples, they suggest new applications for monitoring *in vitro* neuronal differentiation.

We hope that this Research Topic would contribute to the further development of research on Omics Biomarkers in Inflammatory Ocular Diseases.

## Author contributions

MT and MC compiled, drafted, reviewed, and approved. All authors contributed to the article and approved the submitted version.

## Conflict of interest

The authors declare that the research was conducted in the absence of any commercial or financial relationships that could be construed as a potential conflict of interest.

## Publisher's note

All claims expressed in this article are solely those of the authors and do not necessarily represent those of their affiliated organizations, or those of the publisher, the editors and the reviewers. Any product that may be evaluated in this article, or claim that may be made by its manufacturer, is not guaranteed or endorsed by the publisher.
